# Comparative Extracellular Proteomics of *Aeromonas hydrophila* Reveals Iron-Regulated Secreted Proteins as Potential Vaccine Candidates

**DOI:** 10.3389/fimmu.2019.00256

**Published:** 2019-02-18

**Authors:** Yuqian Wang, Xiaoyun Wang, Farman Ali, Zeqi Li, Yuying Fu, Xiaojun Yang, Wenxiong Lin, Xiangmin Lin

**Affiliations:** ^1^Fujian Provincial Key Laboratory of Agroecological Processing and Safety Monitoring, College of Life Sciences, Fujian Agriculture and Forestry University, Fuzhou, China; ^2^Key Laboratory of Crop Ecology and Molecular Physiology, Fujian Agriculture and Forestry University, Fujian Province University, Fuzhou, China

**Keywords:** *Aeromonas hydrophila*, extracellular proteomics, iTRAQ, iron starvation, vaccine candidate

## Abstract

In our previous study, several iron-related outer membrane proteins in *Aeromonas hydrophila*, a serious pathogen of farmed fish, conferred high immunoprotectivity to fish, and were proposed as potential vaccine candidates. However, the protective efficacy of these extracellular proteins against *A. hydrophila* remains largely unknown. Here, we identified secreted proteins that were differentially expressed in *A. hydrophila* LP-2 in response to iron starvation using an iTRAQ-based quantitative proteomics method. We identified 341 proteins, of which 9 were upregulated in response to iron starvation and 24 were downregulated. Many of the differently expressed proteins were associated with protease activity. We confirmed our proteomics results with Western blotting and qPCR. We constructed three mutants by knocking out three genes encoding differentially expressed proteins (Δ*orf01830*, Δ*orf01609*, and Δ*orf03641*). The physiological characteristics of these mutants were investigated. In all these mutant strains, protease activity decreased, and Δ*orf01609*, and Δ*orf01830* were less virulent in zebrafish. This indicated that the proteins encoded by these genes may play important roles in bacterial infection. We next evaluated the immune response provoked by the six iron-related recombinant proteins (ORF01609, ORF01830, ORF01839, ORF02943, ORF03355, and ORF03641) in zebrafish as well as the immunization efficacy of these proteins. Immunization with these proteins significantly increased the zebrafish immune response. In addition, the relative percent survival (RPS) of the immunized zebrafish was 50–80% when challenged with three virulent *A. hydrophila* strains, respectively. Thus, these extracellular secreted proteins might be effective vaccine candidates against *A. hydrophila* infection in fish.

## Introduction

The bacterial species *Aeromonas hydrophila* is an important pathogen of freshwater fish, causing major disease outbreaks and resulting in severe economic losses for the aquaculture industry every year (1). A antibiotics help to control this pathogen and prevent fish disease, but the frequent use of antibiotics might contaminate freshwater ecosystems and increase the spread of antibiotic-resistant bacterial strains (2). It is therefore critical to develop effective immunoprotective vaccines against *A. hydrophila*. It has been suggested that, in carp, mice, and channel catfish, effective vaccine candidates against *A. hydrophila* include DNA and Lipopolysaccharide (LPS) as well as outer membrane, extracellular, and S-layer proteins (3). Much recent research has focused on the immuoprotective properties of extracellular proteins, as these typically affect bacterial virulence (4). For example, in *A. hydrophila*, haemolysin and aerolysin are extracellular proteins that are well-known virulence factors (5). The immunization of catfish with these recombinant proteins significantly increased the relative percent survival (RPS), as compared to unimmunized catfish (6). In addition, two recombinant extracellular proteases (epr2 and epr3) of the *A. hydrophila* strain J-1 conferred significant protection against infection to *Parabramis pekinensis* (7). Therefore, the extracellular proteins of *A. hydrophila* might be good potential candidates for vaccine development.

As high-throughput technology has advanced, proteomics have been frequently used to identify novel antigens for the development of new vaccines (8–10). However, the immuoprotective properties of only a few extracellular or secreted proteins have been characterized to date. This may be in part because many secreted proteins are rare under normal culture conditions (11). This rarity represents a bottleneck for proteome research, despite the current development of highly sensitive mass spectrometry (MS). Thus, it is important to understand the proteomics profiles of the proteins secreted by *A. hydrophila*, which are closer to the natural state of host-pathogen interaction.

Elemental iron is abundant on earth, but ferrous iron, which is necessary for living things, is scarce because of Earth's oxygen-rich atmosphere. Host organisms may limit the ferrous iron concentration in the microenvironment using ferritin, so as to inhibit bacterial growth (12). However, bacteria respond to iron starvation by increasing the iron uptake of their outer membrane proteins (OMPs) or by secreting extracellular proteins to increase virulence and invasion speed (13). Previously, we compared the differential expression of OMPs in response to iron starvation, and identified several OMPs as potential vaccine candidates (14). However, the proteomics profiles of extracellular proteins under iron starvation, and their potential utility in vaccine development, remain largely unclear.

In this study, we compared the expression of extracellular proteins in *A. hydrophila* grown in iron-starved and normal conditions to identify secreted proteins associated with iron-limited environments. After validating the expression of several selected proteins with qPCR and Western blotting, we vaccinated zebrafish with the recombinant candidate proteins, and observed the immune response provoked and the protective efficacy of these proteins. The virulence of several candidate proteins was evaluated by knocking out the encoding genes. Using these techniques, we identified several novel extracellular proteins that may be virulence effectors with a high protective efficacy, making these proteins potential candidates for vaccine development against *A. hydrophila* infection.

## Methods and Materials

### Bacterial Strains and Sample Preparation

The bacterial strain used in this study, *A. hydrophila* LP-2, is a virulent strain that was isolated from a diseased silver carp and was maintained in our laboratory. *A. hydrophila* YT-1 and LP-3 were isolated from diseased *European eel* (*Anguilla anguilla*), which were kindly provided by Prof. Hui Gong from Fujian Academy of Agricultural Sciences (Fuzhou, China) and Dr. Huanying Pang from Guangdong Ocean University (Zhanjiang, China), respectively. *A. hydrophila* ATCC 7966, *Vibrio alginolyticus* ATCC 33787*, Edwardsiella trada* ATCC 15947*Vibrio parahaemolyticus* ATCC 17802*, Vibrio fluvialis* VL 5125*and Edwardsiella trada* EIB202 were kept in our laboratory. The 50% lethal dose (LD50) of *A. hydrophila* LP-2, LP-3 and YT-1 in zebrafish were 8.1 × 10^3^, 6.5 × 10^6^, and 1.2 × 10^7^ cells, respectively, suggesting that these strains are virulent.

To isolate extracellular proteins, *A. hydrophila* LP-2 was streaked for isolation on Luria-Bertani (LB) agar medium and incubated overnight at 30°C, as previously described (15). One colony was transferred to 5 mL fresh LB medium overnight, and then diluted 1:100 in 100 mL LB with or without 150 μM 2,2′-dipyridyl (DIP). The resulting mixtures were incubated at 30°C with shaking at 200 rpm for about 5 h (until OD at 600 nm was ~1.5). Samples were collected by centrifugation at 4500 × g for 20 min at 4°C, and each supernatant was passed through a 0.45-μm filter membrane and then a 0.22-μm filter membrane to remove all of the bacterial cells. We then added 8% (v/v) ice-cold trichloroacetic acid to all of the filtered supernatants and incubated the resulting mixtures at 4°C overnight. After centrifugation at 11,000 × g for 40 min at 4°C, the resultant pellet was washed with pre-cooled acetone three times. Pellets were dissolved in 8 M urea, and protein concentrations were determined by the Bradford method. Dissolved pellets were stored at −80°C.

### Protein Identification Using iTRAQ Labeling and LC-MS/MS

Protein digestion and iTRAQ labeling were performed as previously described (16). The iTRAQ labeling scheme was as follows: the two untreated biological repeats (controls) were labeled 113 and 117, and the two biological repeats treated with 150 μM DIP were labeled 114 and 118. Labeled peptides were quantified with an AB Sciex TripleTOF 5,600 mass spectrometer (AB SCIEX; Concord, ON, Canada) equipped with a NanoAcquity UPLC system (Waters, Milford, MA, USA). All of the settings were as previously described (17), except that we used the LP-2 genome re-sequencing database produced in our laboratory (unpublished data).Proteins matching at least two unique peptides, with a false discovery rate (FDR) < 1%, were considered for further analysis. To quantify protein expression, we compared the degree of change in the iTRAQ ratio of the samples treated with DIP to that of the untreated controls. If the fold change as compared to the controls was >1.5 or <0.667 (a fold change of ±1.5), and the *p* < 0.05 for both biological replicates, these proteins were deemed significantly differentially expressed.

### Bioinformatics Analysis

We investigated the gene ontology (GO) and Kyoto Encyclopedia of Genes and Genomes (KEGG) of the differentially expressed proteins using the online omicsbean resource (http://www.omicsbean.cn/) (18). The generated histogram and volcano plots were analyzed in R software version 3.4.0.

### Purification of Recombinant Proteins

Six genes (*orf01609, orf01830, orf01839, orf02943, orf03355*, and *orf03641*) were cloned into the pET-32a plasmid and transformed into *Escherichia coli* BL21(DE3), using the primer sequences listed in Supplementary Table 1. The recombinant proteins were purified as previously described (19). Briefly, the recombinant strain was incubated in 5 mL LB medium overnight at 37°C 200 rpm, transferred to 200 mL LB medium supplemented 1:100 with 100 μg/mL ampicillin (Amp) for 2–3 h. Next, 0.5 mM Isopropyl -β-D-thiogalactopyranoside (IPTG) was added to the ice-cold culture for protein induction at 16°C for about 6–8 h. After washing with phosphate-buffered saline (PBS) three times, bacterial pellets were resuspended in binding buffer (25 mM Na_2_HPO_4_·12H_2_O, 10 mM NaH_2_PO_4_·2H_2_O, 500 mM NaCl, and 5 mM imidazole). The suspension was sonicated on ice for 30 min, and centrifuged to remove cellular debris. Finally, the supernatants containing the fusion proteins were purified using affinity chromatography on a HisTrap Sepharose nickel column (GE Healthcare, Uppsala, Sweden).

### qPCR Assay

To measure the expression of genes associated with immune response in vaccinated fish, we collected tissue samples from zebrafish haslets (four fish per group). Tissue samples were frozen in liquid nitrogen and then ground. RNA was extracted from each tissue sample using RNAiso Plus reagent (TaKaRa Bio, Tokyo, Japan) as previously described (20). Briefly, RNA quality was first evaluated with a NanoDrop One (Thermo Scientific, USA), and then 1 μg RNA from each tissue sample was reverse-transcribed into cDNA using a PrimeScript RT reagent Kit (Takara Shuzo, Otsu, Japan). Real-time PCR was performed with the CFX96 Touch Deep Well Real-Time PCR Detection System (Bio-Rad, USA) using SYBR Premix Ex Taq II Kits (Takara Shuzo, Otsu, Japan), following the manufacturer's instructions. The β-actin gene was used as the internal control.

To validate gene expression under iron-limited conditions, total RNA was extracted from *A. hydrophila* LP-2 cells grown with and without 150 μM DIP. qPCR assays were performed as above, using the *16S rRNA* gene as the internal control. All the qPCR primers used in this study are listed in Supplementary Table 2. Three independent biological repeats were performed for DIP each experiment.

### Western Blotting

The recombinant proteins were purified, and injected into mice to produce specific polyclonal antibodies. Western blotting was then performed as previously described (21). Briefly, a 12% SDS-PAGE was performed to separate the extracellular proteins grown with or without 150 mM DIP. After electrophoresis, proteins were transferred to polyvinylidene fluoride (PVDF) membranes in transfer buffer with a Trans-Blot Turbo Transfer System (Bio-Rad, Hercules, CA, USA). Membranes were blocked with PBST containing 5% (w/v) non-fat milk for 1 h, and then incubated with diluted mouse antiserum as the primary antibody. After being washed three times with PBST, membranes were incubated with horseradish peroxidase conjugated goat anti-mouse antibody (1:4,000) as the secondary antibody. The immune-stained proteins were detected using Clarity Western ECL Substrate (Bio-Rad) and visualized with Image Lab software (Bio-Rad) on the ChemiDoc MP imaging system (Bio-Rad, Hercules, CA). Finally, PVDF membranes were stained with Coomassie R-350 to verify that the loading amounts were equal.

### Construction of Deletion Mutants

We constructed deletion mutants by deleting three of the six genes of interest (*orf01609, orf01830*, and *orf03641*) as previously described (22). Briefly, we amplified the sequence fragments (~500 base pairs (bp) length) flanking each target gene ORF in *A. hydrophila* LP-2, using the primers listed in Supplementary Table 3. The resulting PCR products were ligated into the suicide vector pRE112 (Cm^r^). The reconstruction plasmids were transformed into *E. coli* MC1061 λ*pir* competent cells and screened on LB agar containing 30 μg/ml chloramphenicol (Cm). Positive plasmids were transformed into *E. coli* S17-1 λpir competent cells and introduced into *A. hydrophila* LP-2 via bacterial conjugation. Single crossover mutants were screened on LB agar containing 100 μg/ml ampicillin and 30 μg/ml Cm for the first homologous recombination. Positive colonies were transferred into 1 ml fresh LB incubated overnight and then cultured on 20% sucrose LB ager plates for the second homologous recombination. Finally, deletion mutants were verified using PCR and sequencing using primers P7 and P8 (Supplementary Table 1).

### Characterization of Mutant Strains

The phenotypes of the mutant strains were characterized by hemolytic activity, extracellular protease (ECPase) activity, and biofilm formation. Hemolytic and ECPase activity were assayed as previously described (23, 24). Biofilm formation was measured using crystal violet staining (25).

### Bacterial Challenge of Mutant Strains

Zebrafish were purchased from the Fuzhou Flower, Bird & Fish Market (Fuzhou, China) and challenged by widetype and mutant strains of *A. hydrophila* as previously described (26). Briefly, sixteen zebrafish per treatment group were injected intraperitoneally with 10 μL *A. hydrophila* LP-2 (WT) or with 10 μL *A. hydrophila* mutants at a density of 10^3^-10^5^ CFU/ml PBS. Negative control zebrafish were injected with 10 μL sterile PBS. Challenged fish were monitored for 14 days, and LD50 values were calculated using the statistical approach of Reed and Muench (27). For the livability investigation of mutant strains, the zebrafish were intraperitoneally injected with 10 μl 2.0 × 10^4^ cfu/ml mutants, and observed for 14 days as previously described with modification (26). The kidneys of dead fish were collected and were homogenized in 1 ml PBS. The homogenates were serially diluted and plated onto RS plates (Hope Bio Co.,Ltd., Qingdao, China) and incubated at 30°C for 16 h. Finally, the positive colonies were verified by PCR and sequencing.

### Vaccination

Zebrafish were randomly divided into 7 groups with 20 fish per group, as previously described with some modifications (28). In brief, about 3 μg purified fusion protein emulsified with incomplete Freund's adjuvant (IFA) was administered to each fish via intraperitoneal injection. Negative control fish were injected with an equal amount of bovine serum albumin (BSA) which emulsified with IFA. After 28 days injection, fish in each group were challenged with 1.6 × 10^5^ CFU/ml *A. hydrophila* LP-2 in 10 μL sterile PBS, delivered via intraperitoneal injection. This dose was 20-fold greater than the LD50 of *A. hydrophila* LP-2. All of the fish were observed for 14 days, and cumulative mortality and clinical signs were recorded daily. The experiment was repeated three times independently.

## Results

### Identification of Differentially Expressed Proteins in Response to Iron Starvation Using iTRAQ Technology Coupled With MS

**Figure 1:**
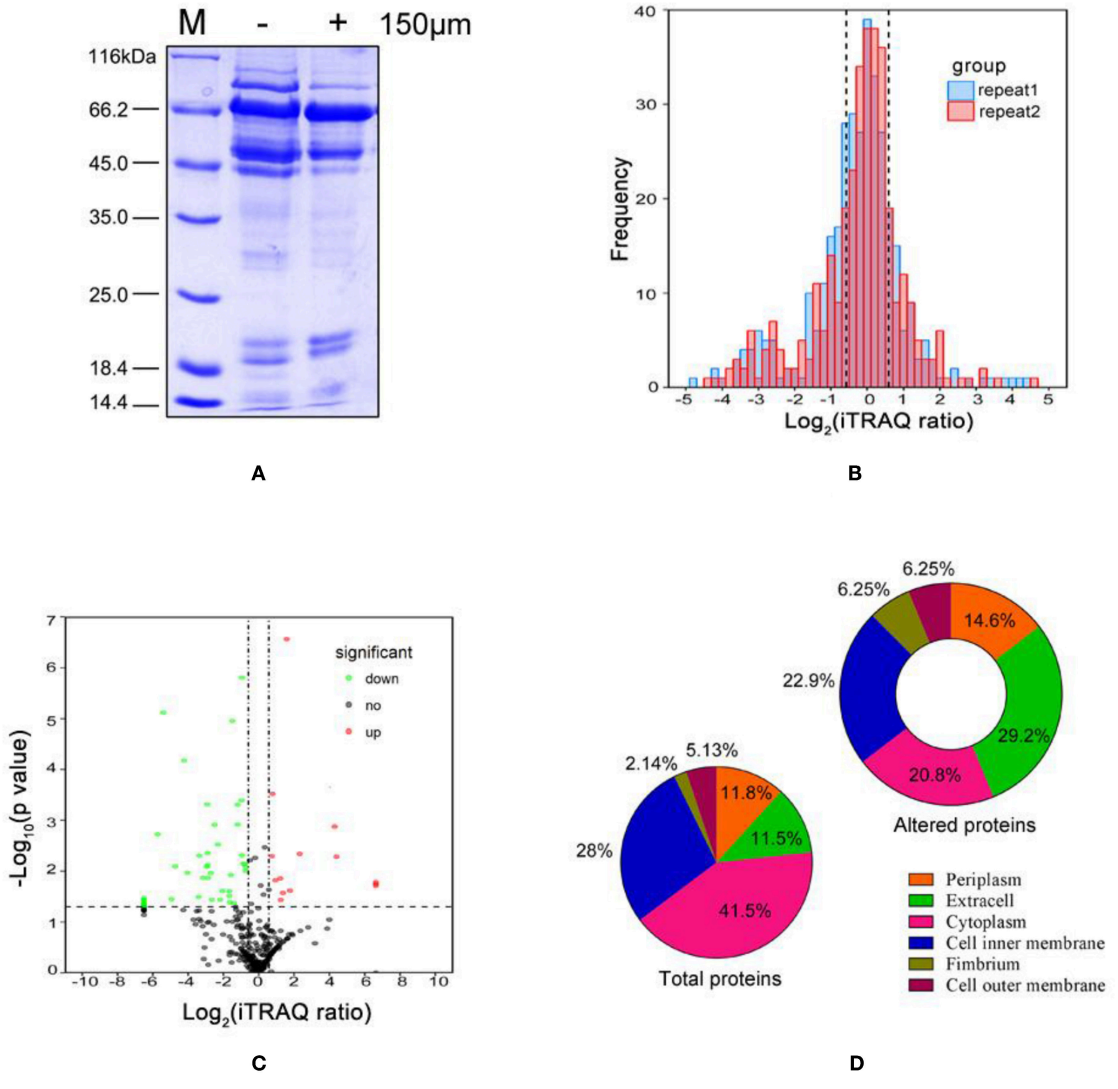
Characteristics of *Aeromonas hydrophila* LP-2 grown in iron-limited conditions. **(A)** Coomassie brilliant blue(CBB) stained SDS-PAGE of extracelluar protein fractions from *A. hydrophila* treated with 150 μM DIP. Lane M, molecular mass standards. **(B)** The frequency distribution of protein iTRAQ log_2_ ratios between the two biological replicates. **(C)** Volcano plot comparing the extracellular proteomics of *A. hydrophila* grown in iron-limited conditions and *A. hydrophila* grown in normal conditions. Solid lines indicate statistically significant differences in protein expression (iTRAQ ratio >1.5 or <0.66 and *p* < 0.05 for both biological replicates). Upregulated proteins are indicated by red circles; downregulated proteins are indicated by green circles; and unaltered proteins are indicated by black circles. **(D)** Subcellular locations predicted for all identified proteins and for all differentially expressed proteins.

Extracellular proteins differentially expressed in *A. hydrophila* LP-2 grown with or without DIP treatment were isolated and separated using SDS-PAGE (Figure 1A). We observed that the different treatments resulted in different bands, which indicated that the extracellular proteins might be involved in iron homeostasis. However, the specific proteins could not be identified due to the low resolution of SDS-PAGE. Thus, we used iTRAQ labeling coupled with MS proteomics to quantify the differences in extracellular protein expression. We identified 341 differentially expressed proteins (Supplementary Table 4), with a substantial conservative threshold (confidence level ≥ 95%). Most of the ratios of these comparison were normally distributed (log_2_ scale range: ±1) across both biological duplicates (Figure 1B). Under iron-limited conditions, nine proteins were significantly upregulated and 24 proteins were significantly downregulated (*p* < 0.05), with a minimum ± 1.5-fold change in expression as compared to controls (Table 1 and Figure 1C). Subcellular localization prediction indicated that most of the 341 differentially expressed proteins were located in the cytoplasm, the inner membrane, and the periplasm (Figure 1D). Moreover, we found the extracellular proteins proportion increased almost 3-folds from 11.5 to 29.2% in 33 altered proteins than total identified proteins components, which suggest the important role of secreted protein in iron starvation condition.

**Table 1:** Selected identification of differentially proteins of *A. hydrophila* LP-2 in iron starvation conditions by iTRAQ labeling analysis.

**Accession**	**Protein description**	**Peptide matched**	**%Coverage**	**iTRAQ ratio (Repeat1)**	***P*-Value (Repeat1)**	**iTRAQ ratio (Repeat2)**	***P*-Value (Repeat2)**	**Homologous to ATCC7966**
ORF02943	Hypothetical protein	12	25.94	21.28	5.24E-03	58.08	4.31E-04	AHA_1448
ORF01839	Periplasmic iron-binding protein FecB	19	40.13	19.59	1.32E-03	41.30	1.31E-02	AHA_1964
ORF01830	FIG00919923: hypothetical protein	93	56.61	10.19	5.96E-12	9.82	3.70E-03	AHA_2481
ORF01609	Putative extracellular serine protease	3866	97.6	2.99	2.73E-07	4.06	3.79E-08	AHA_2687
ORF02793	Periplasmic molybdenum-binding protein ModA	18	43.8	2.33	1.40E-02	3.66	6.64E-03	modA
ORF04443	Outer membrane vitamin B12 receptor BtuB	21	40.74	2.58	2.69E-02	2.36	1.93E-02	hgpB
ORF00614	FIG003551: hypothetical protein	16	53.96	2.36	3.68E-02	1.84	2.88E-02	AHA_3660
ORF03355	FIG00545237: hypothetical protein	40	69.86	1.66	5.06E-03	1.98	3.45E-04	AHA_1098
ORF03641	Extracellular zinc protease	232	64.69	1.71	3.04E-04	1.91	3.43E-06	AHA_0851
ORF04406	Putative lipase	99	46.77	0.54	7.20E-03	0.52	3.82E-02	AHA_0104
ORF03115	Sialidase	174	74.79	0.51	4.07E-04	0.52	2.73E-02	nanH*
ORF03984	Microbial collagenase, secreted	185	68.09	0.36	1.10E-05	0.58	3.44E-03	AHA_0517
ORF02720	Substrate binding periplasmic protein MalE	63	66.16	0.43	4.95E-04	0.34	2.03E-02	AHA_1667
ORF03869	Type II secretory pathway, pullulanase	118	58.08	0.20	3.02E-03	0.49	7.19E-03	AHA_0628
ORF01696	Alanine racemase	11	33.09	0.13	8.19E-03	0.44	2.38E-02	alr-3
ORF02315	6-phosphofructokinase	9	29.1	0.10	4.93E-03	0.45	2.16E-02	pfkA
ORF04420	Porphobilinogen synthase	15	42.18	0.24	2.45E-02	0.24	3.19E-02	hemB
ORF00636	tolB protein precursor	6	22.9	0.22	3.64E-02	0.18	3.05E-02	tolB
ORF03513	Chitinase	204	60.07	0.18	1.23E-03	0.20	2.21E-05	AHA_0977
ORF04186	SSU ribosomal protein S13p	5	37.29	0.14	7.62E-03	0.20	1.18E-02	rpsM
ORF00322	FIG00362058: hypothetical protein	21	61.02	0.14	1.35E-02	0.18	2.27E-02	AHA_3948
ORF02209	FabA	6	42.47	0.15	1.10E-02	0.15	1.90E-02	fabA
ORF03511	Chitinase	187	72.97	0.13	4.82E-04	0.16	2.88E-04	AHA_0979
ORF03029	hypothetical protein	37	30.65	0.11	1.33E-02	0.14	2.99E-02	AHA_1406
ORF02740	Glycerol kinase	5	14.15	0.06	1.07E-02	0.09	1.85E-02	glpK
ORF03889	Chitin binding protein	360	78.69	0.05	6.62E-05	0.09	1.04E-04	AHA_0610
ORF01532	Cytochrome c4	29	46.23	0.03	3.55E-02	0.06	4.50E-02	AHA_2763
ORF02296	Chitinase	218	80.29	0.02	7.62E-06	0.05	3.47E-06	AHA_2363
ORF01386	Cold shock protein CspE	5	50	0.02	1.88E-03	0.03	8.51E-03	AHA_2864
ORF04175	CopG protein	2	12.59	0.01	4.48E-02	0.01	4.30E-02	AHA_0347
ORF02395	T1SS secreted agglutinin RTX	5	9.742	0.01	4.51E-02	0.01	4.39E-02	AHA_2463
ORF01538	Peptidyl-prolyl cis-trans isomerase PpiB	6	39.39	0.01	3.72E-02	0.01	3.70E-02	ppiB
ORF01093	Peptidyl-tRNA hydrolase	2	32.83	0.01	4.84E-02	0.01	4.83E-02	pth

* *homologous to A. hydrophila strain ATCC 39315*.

### Functional Classification and Annotation of Differentially Expressed Proteins

**Figure 2:**
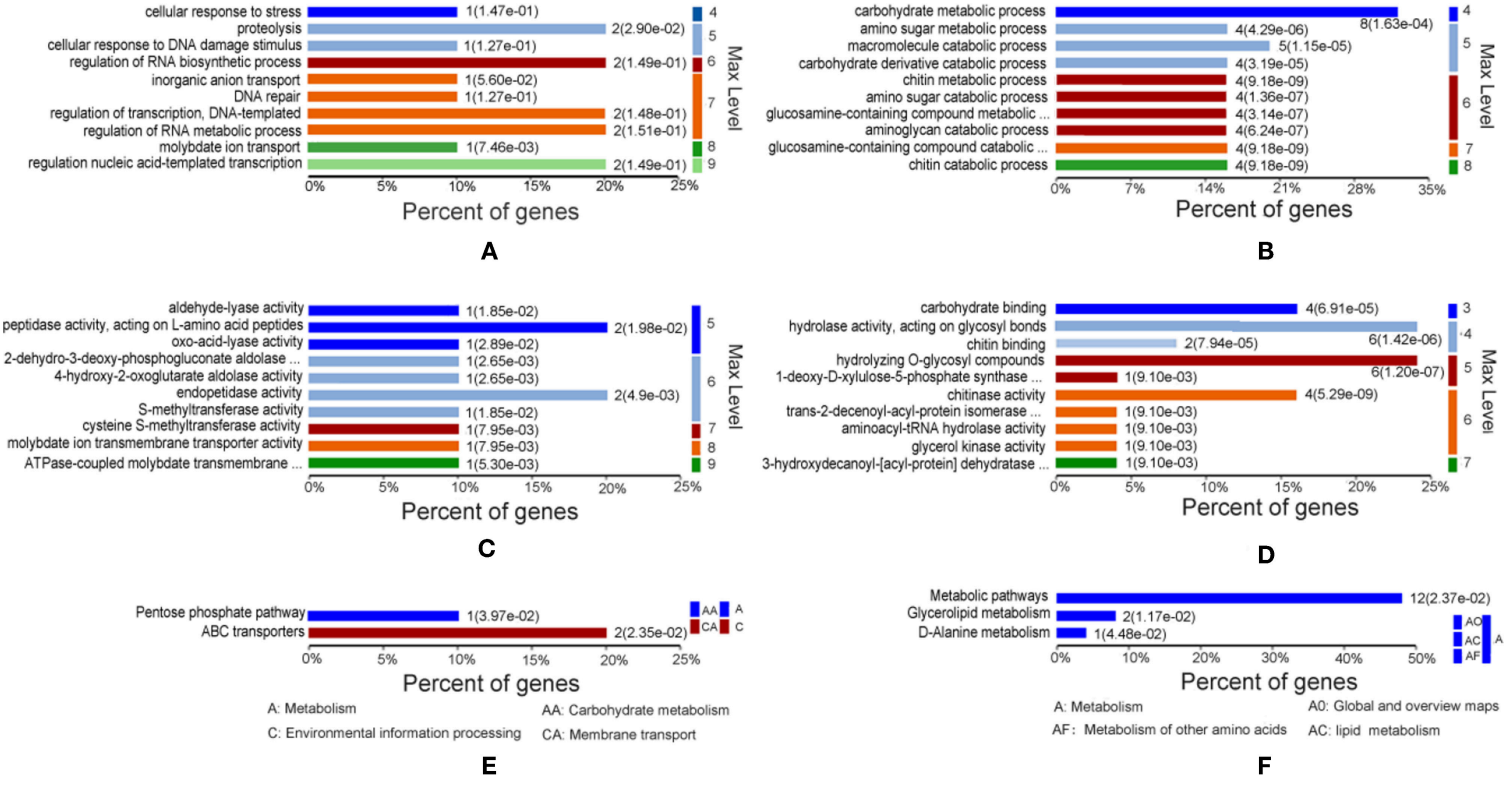
Gene Ontology (GO) of differentially expressed proteins under iron starvation. **(A,C,E)** Upregulated proteins associated with biological processes, molecular functions, and KEGG pathways, respectively. **(B,D,F)** Downregulated proteins associated with biological processes, molecular functions, and KEGG pathways, respectively. The *p-*value cutoff was set at < 0.05. Different colors represent different GO levels, from 3 to 9.

To investigate the functional characteristics of the proteins differentially expressed in *A. hydrophila* LP-2 starved of iron, we clustered these proteins by GO using the homologous *A. hydrophila* ATCC 7966 genome database. Most proteins were enriched in biological processes, molecular functions, and KEGG pathways (Figure 2). In general, under iron-starvation, upregulated proteins were enriched in fewer GO clusters than downregulated proteins. Proteolysis, regulation of nucleic acid-template transcription, and RNA biosynthesis regulation were the three biological process most enriched in upregulated proteins, while carbohydrate metabolism, amino sugar metabolic processes, and macromolecule catabolic processes were the biological processes most enriched in downregulated proteins (Figures 2A,B). The molecular function analysis indicated that the upregulated proteins were related to the activity of several different proteases, including aldehydelyase, peptidase, S-methyltransferase, and ATPase coupled molybdate. In contrast, the downregulated proteins were involved in processes including carbohydrate binding, hydrolase activity, and chitinase activity (Figures 2C,D). The KEGG pathways most enriched in upregulated proteins included the pentose phosphate pathway and the ATP-binding cassette (ABC) transporters, while the KEGG pathways most enriched in downregulated proteins included metabolic pathways, glyceroloid metabolism, and D-alanine metabolism (Figures 2E,F).

### Predicted Protein-Protein Interactions (PPIs) Among Extracellular Proteins in Response to Iron Starvation

**Figure 3:**
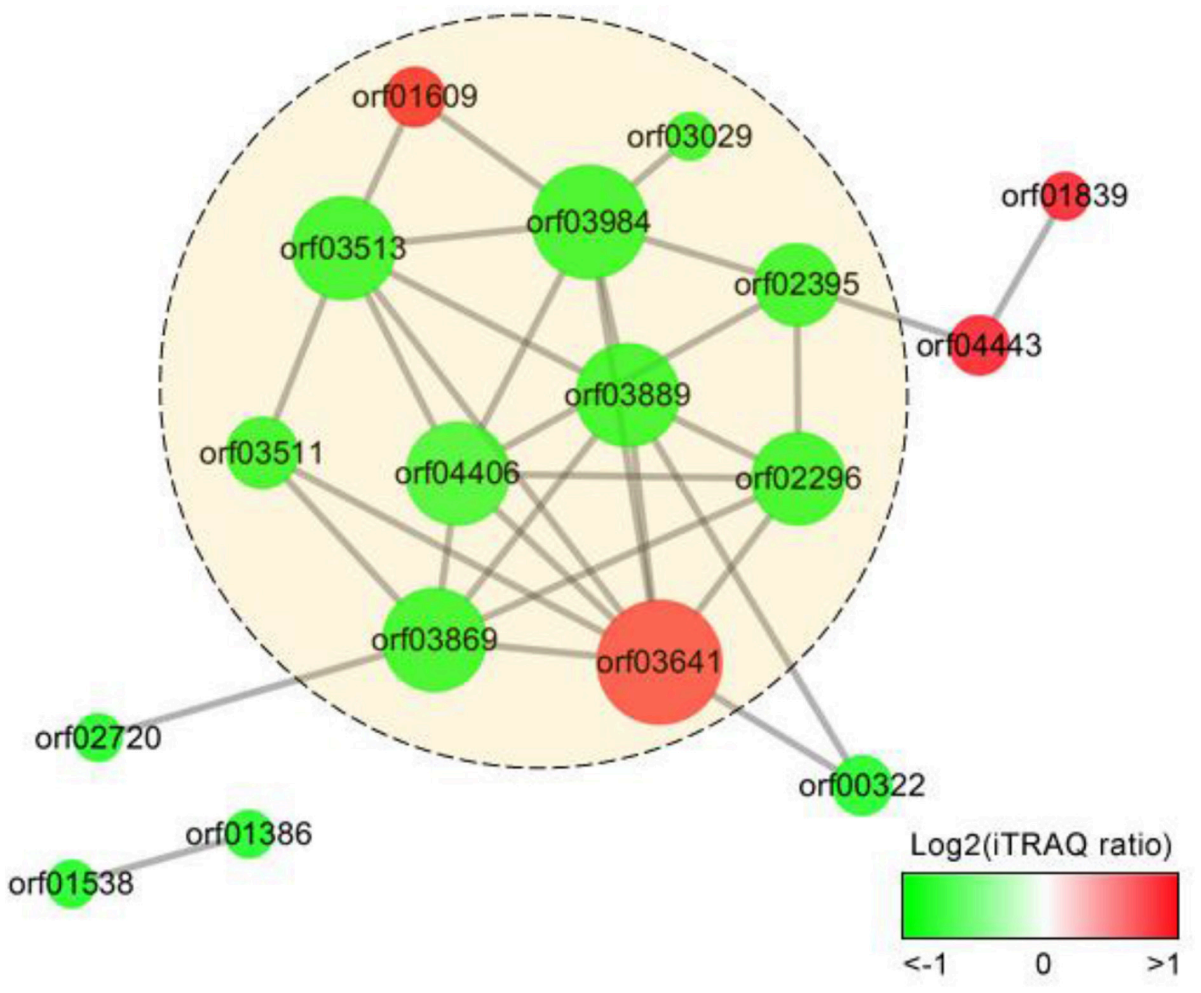
Protein-protein interaction (PPI) networks of the extracellular proteins differentially expressed in *A. hydrophila* under iron starvation condition. The gradient color indicates iTRAQ protein ratio (on a log_2_ scale), and size represents protein interaction frequency. Predicted extracellular proteins are displayed in yellow circles.

We analyzed the PPIs of the proteins differentially expressed in response to iron starvation in *A. hydrophila* LP-2 based on the homologous *A. hydrophila* ATCC 7966 genomic database. The PPI network we constructed included nine genes encoding down-regulated proteins (*orf03029, orf02395, orf03889, orf02296, orf03869, orf04406, orf03984, orf03511*, and *orf03513*) and two genes encoding up-regulated proteins (*orf01609* and *orf03641*) (Figure 3). Interestingly, *orf03984* and *orf03641* were important nodes (hubs): *orf03984* interacted with seven other differentially expressed protein, and *orf03641* interacted with eight other differentially expressed proteins. Some periplasmic and outer cell membrane proteins were also included in our PPI network: two upregulated (*orf04443*(*hgpB)* and *orf01839*) and four downregulated [*orf02720, orf01386, orf00322*, and *orf01538(ppiB*)]. Our results suggested that secreted proteins have many biological functions and are involved in complex protein-protein interactions during iron homeostasis.

### Validation of the Proteomics Results in Protein Level

**Figure 4:**
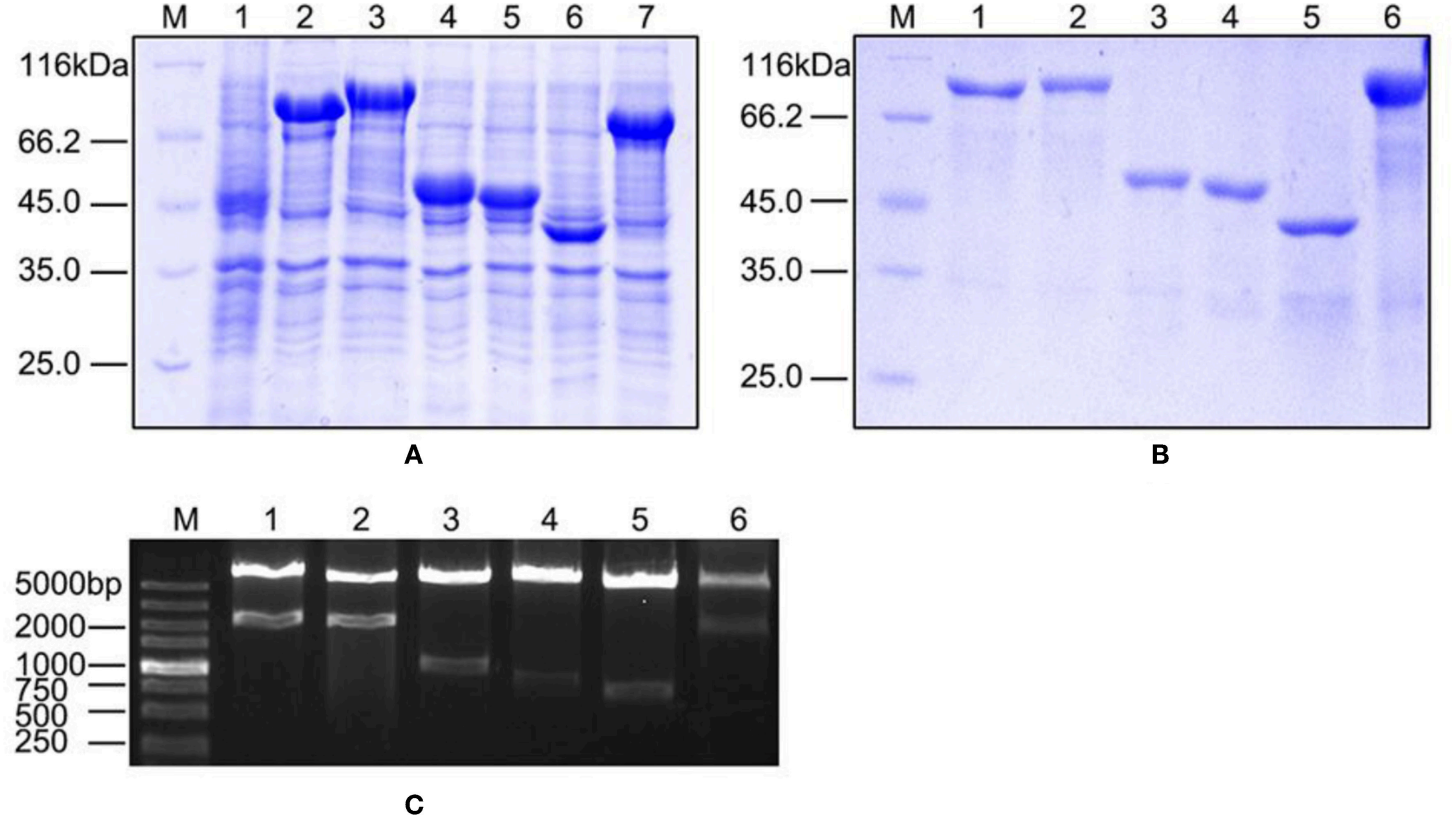
The characteristics of overexpressed and purified recombinant proteins. **(A)** SDS-PAGE of overexpressed proteins in the BL21 strain. Lane 1: negative control; Lanes 2–7: ORF01609, ORF01830, ORF01839, ORF02943, ORF03355, and ORF03641, respectively. **(B)** SDS-PAGE of purified proteins. Lanes 1–6: purified ORF01609, ORF01830, ORF01839, ORF02943, ORF03355, and ORF03641, respectively. **(C)** Restriction enzyme identification of recombinant extracellular protein plasmids carried by the pET-32a vector. M: DL5000 marker; Lanes 1–6: ORF01609, ORF01830, ORF01839, ORF02943, ORF03355, and ORF03641, respectively.

We validated our proteomics data at the protein level using western blots. First, we cloned six genes (*orf01609, orf01830, orf01839, orf02943, orf03355*, and *orf03641*), all of which encode proteins that were upregulated under iron limiting conditions based on our proteomics analysis. These genes were overexpressed to produce purified recombinant proteins (Figures 4A,B). Recombinant plasmids were further validated using restriction enzymes (Figure 4C), to ensure that the correct recombinations had been constructed.

**Figure 5:**
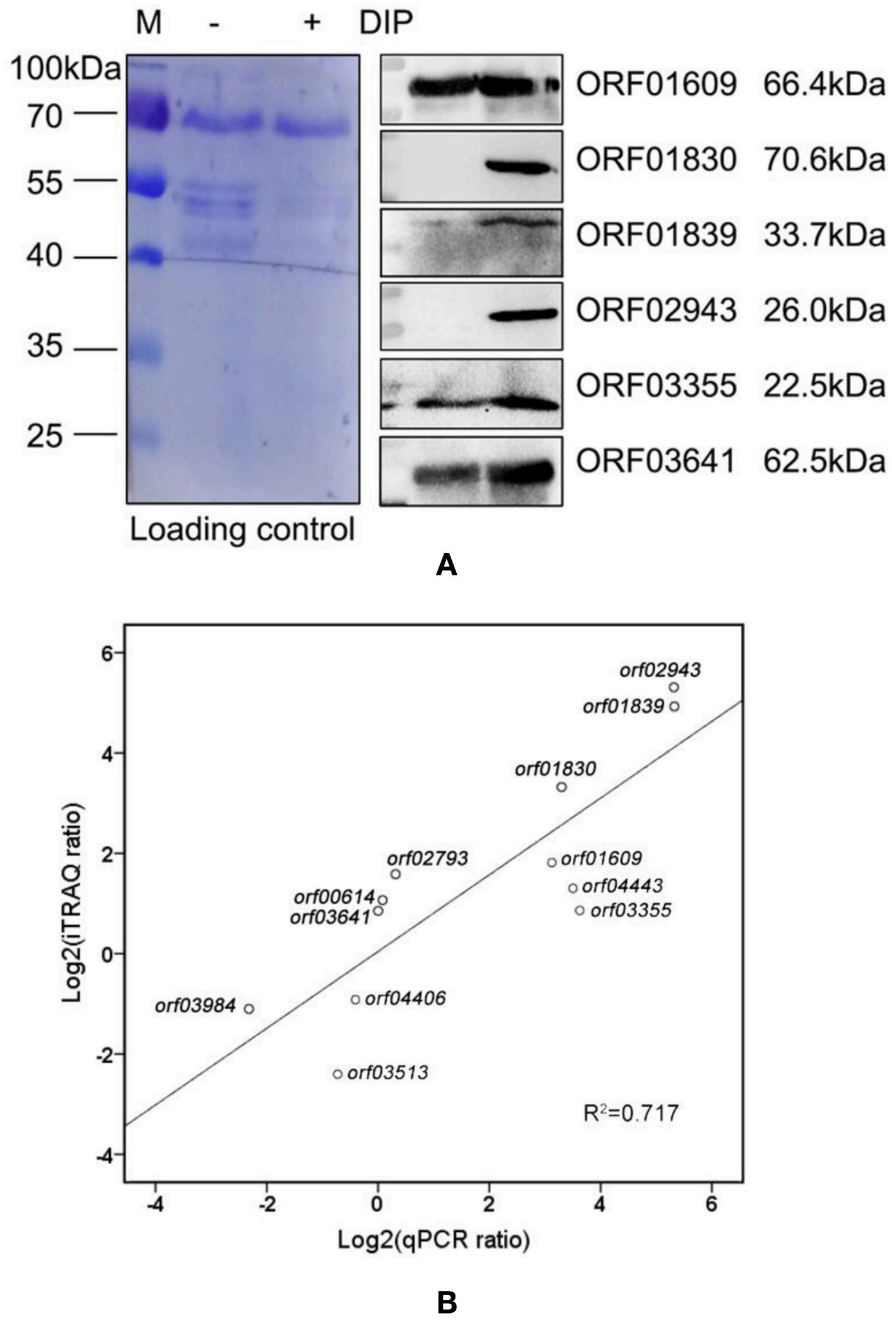
Correlations between selected genes and proteins. **(A)** Western blot of differentially expressed extracellular proteins in *A. hydrophila* LP-2 with and without DIP treatment. Coomassie R-350 stained on PVDF membrane was used as the loading control. **(B)** The correlation between mRNA expression and proteomics in nine upregulated genes *(orf00614, orf01609, orf01830, orf01839, orf02793, orf02943, orf03355, orf03641*, and *orf04443*), and three downregulated genes (*orf03513, orf03984*, and *orf04406*).

Mice were immunized with the purified recombinant proteins via subcutaneous injection to obtain specific polyclone antibodies. Western blotting was performed to validate our proteomics analysis. The six selected proteins were significantly upregulated in groups treated with DIP as compared to the control group (Figure 5A), especially ORF01830, ORF01839, and ORF02943. These three proteins were undetectable in the control group, but sharply upregulated under iron limited conditions. These results indicated that our proteomics results were reliable. Our results also suggested that these iron-related extracellular proteins might play a role in bacterial virulence, and might be potential vaccine candidates.

### Validation of the Proteomics Results at the mRNA Level

To further confirm our proteomics results, we used qPCR to measure the expression levels of selected genes indicated to encode differentially expressed proteins by our proteomics analysis. There was a high correlation (*r*^2^ = 0.717) between mRNA and proteomics across 12 genes of interest (Figure 5B). Except for *orf00614, orf03641*, and *orf02793*, the mRNA expression levels of the selected genes were consistent with our proteomics results. Although the intrinsic mechanism of the discrepancy between transcription and protein level remains largely unknown because of complicated regulative networks, the difference in temporal dimensions, time regulation delay, and protein post-translational modifications may contribute to the difference in both levels (29). Those results indicate that the combination of validation between protein and mRNA level will provide clearer clues for bacterial behaviors in iron starvation.

### Deletion of Genes Encoding Extracellular Proteins and Characterization of Physiological Function

**Figure 6:**
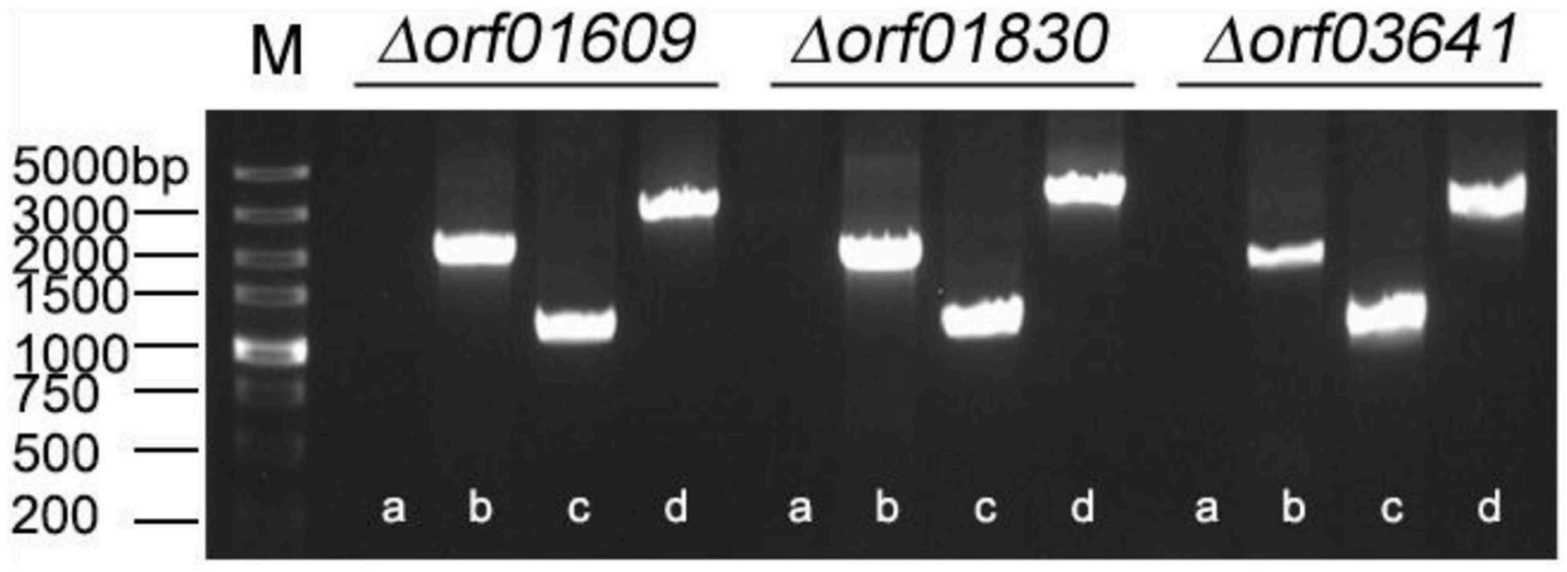
Confirmation of the knockout mutant strains Δ*orf01609*, Δ*orf01830*, and Δ*orf03641*. M: DL5000 marker; a: The fragment of genomic deletion mutant DNA amplified using cloning primers. b: The fragment of genomic wild-type (LP-2) DNA amplified using cloning primers. c: The fragment of genomic deletion mutant DNA amplified using the primer pair P7/P8. d: The fragment of genomic wild-type (LP-2) DNA amplified using the primer pair P7/P8.

We next investigated the biological functions of the target proteins. Three of the six selected genes (*orf01609, orf01830*, and *orf03641*) were successfully knocked out using traceless methods (Figure 6). The physiological functions of these genes included biofilm formation, protease activity, and hemolysis. The LD50s of these knockout strains were compared with wildtype *A. hydrophila* LP-2 in zebrafish (Table 2). Although biofilm formations of mutants were unaffected, their protease activities were significantly decreased. The hemolysis activity had no difference in Δ*orf01609* and Δ*orf01830* when compared to WT control, while significantly increased in Δ*orf03641*. Meanwhile, to better understand the role of targeted genes in the virulence of *A. hydrophila* LP-2, zebrafish were injected intraperitoneally with WT and mutant strains. Mortality was recorded regularly for 14 days following infection. We found that the LD50s of the Δ*orf01609* (3.30 x 10^4^ CFU/ml) and Δ*orf01830* (2.90 × 10^4^ CFU/ml) mutants were about 4- and 2-fold greater, respectively, than that of *A. hydrophila* LP-2 (0.81 × 10^4^ CFU/ml), indicating a substantial reduction in virulence. However, the LD50 of Δ*orf03641* (1.35 × 10^4^ CFU/ml) was similar to that of *A. hydrophila* LP-2. Our results therefore indicated that *orf01609* and *orf01830* may be novel virulent effectors, and might play important roles in bacterial invasions.

**Table 2:** Characteristics of the mutant strains.

**Characteristics**	***A. hydrophila* LP-2**	**Δ*ORF01609***	**Δ*ORF01830***	**Δ*ORF03641***
Biofilm formation^a^	0.84 ± 0.06	0.84 ± 0.12	0.83 ± 0.07	0.80 ± 0.12
Activity of ECPase (A_442_)^b^	0.34 ± 0.01	0.27 ± 0.01**	0.30 ± 0.02*	0.26 ± 0.01**
Hemolysis (%)^c^	4.27 ± 0.11	4.76 ± 0.19	4.01 ± 0.32	12.87 ± 0.18**
LD50 (cfu/mL)^d^	0.81 × 10^4^	3.30 × 10^4^**	2.90 × 10^4^**	1.35 × 10^4^

*Values are the mean ± standard deviation of three independent trials. Significant differences between A. hydrophila LP-2 and each mutant strain are indicated by asterisks*.

* P < 0.05;

** *P < 0.01*.

a *96-well-polypropylene plates incubated with bacteria for 48 h at 30°C and was measured using crystal violet staining method*.

b *bacteria were cultured on 1.5% LB agar plates and incubated for 18 h at 30°C, then collected using PBS. And use azocasein assay for extracellular protease*.

c *extracellular protease hemolytic activity was measured using defibrinated sheep blood. Culture supernatant was incubated with blood in PBS for 1 h at room temperature*.

d *LD50s were evaluated in healthy zebrafish with the weight of 0.32 ± 0.06 g*.

### mRNA Expression of Immune-Related Genes in Response to Recombinant Proteins, as Measured by qPCR

**Figure 7:**
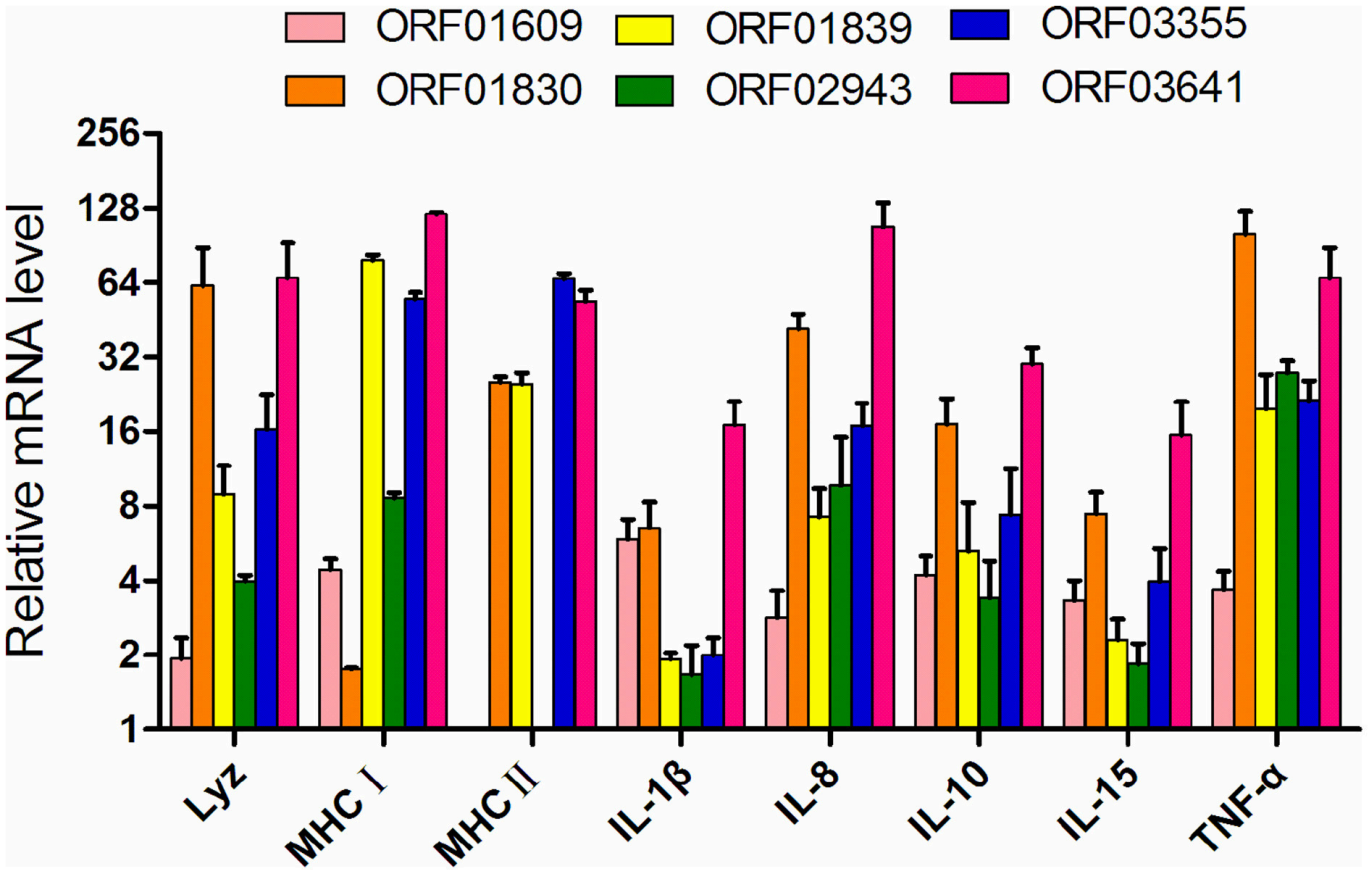
Expression of immune-related genes in zebrafish immunized with recombinant proteins as compared to controls treated with BSA, as quantified by qPCR. Internal organs were collected before the *A. hydrophila* LP-2 challenge.

The six iron-related recombinant proteins were injected into zebrafish. The immune response of the zebrafish was then evaluated using qPCR based on the transcription of eight immune-related genes (Lyz, MHC I, MHC II, IL-1β, IL8, IL10 IL15, and TNF-α) with BSA immunization as negative control. After ORF02943 and ORF01609 immunization, MHC II expression decreased slightly. And IL-1β expression was not significantly different after ORF02943 immunization (Figure 7), However, the expression levels of most of the immune-related genes increased significantly (≥1.5-fold) after immunization with the recombinant proteins. Therefore, these recombinant proteins could stimulate the immune response in fish, and might be good candidates for an *A. hydrophila* vaccine.

### Protective Efficacy of Iron-Related Recombinant Proteins

**Figure 8:**
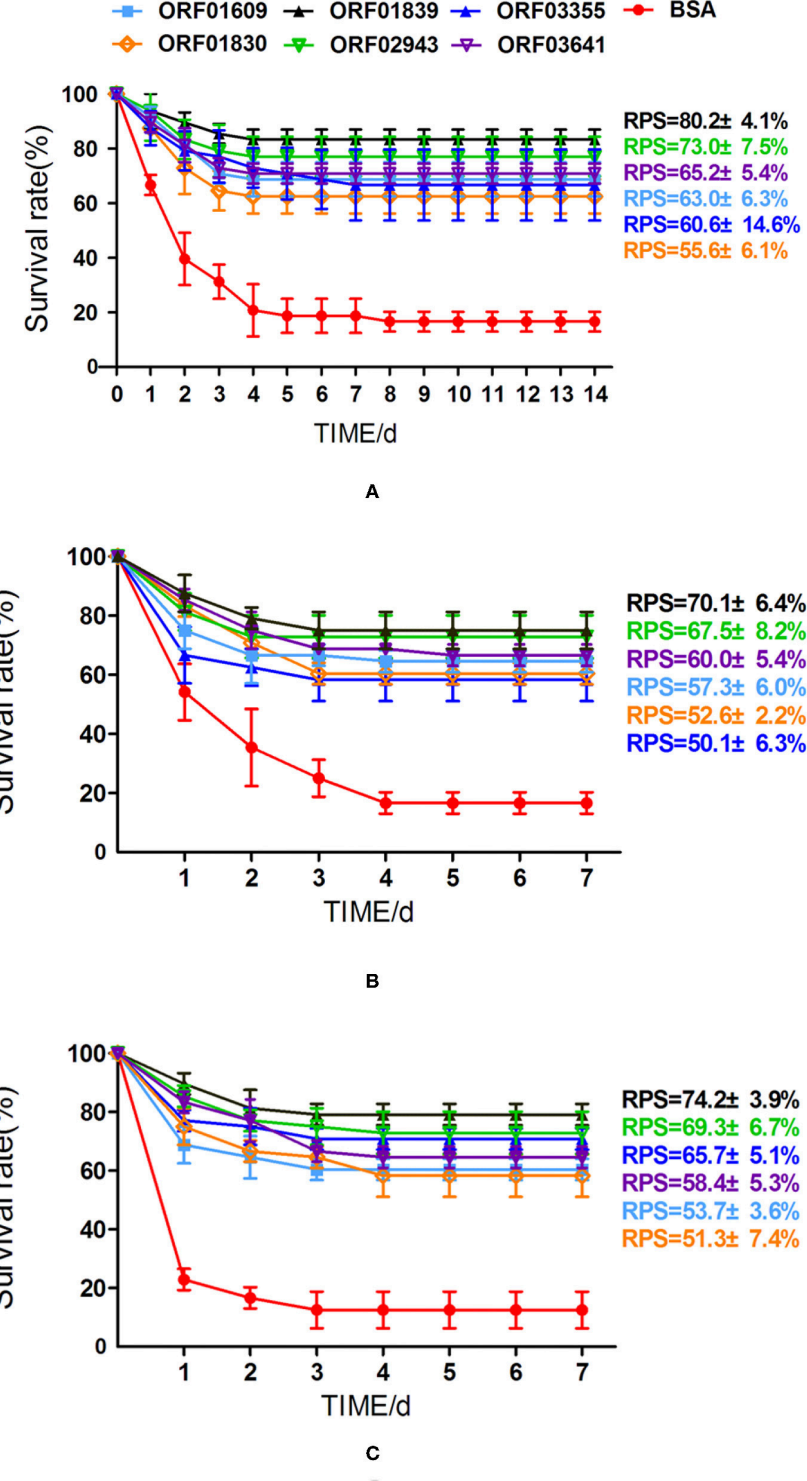
Cumulative survival rates of zebrafish after challenge with *A. hydrophila*. **(A)** Zebrafish vaccinated with six iron-related extracellular proteins (ORF01609, ORF01830, ORF01839, ORF02943, ORF03355, and ORF03641) and a control treated with BSA were challenged with *A. hydrophila* LP-2 for 14 days. **(B,C)** Zebrafish vaccinated with six recombinant proteins and a control treated with BSA were challenged with *A. hydrophila* LP-3 and YT-1 for 7 days, respectively. Mortalities were recorded for 14 days. Data represent the mean ± SEM of *n* ≥ two independent replicates.

To better understand the immunoprotective efficacy of proteins that were upregulated during iron starvation, zebrafish were immunized with related recombinant proteins (or with BSA, as the control) over 28 days. Immunized zebrafish were then challenged with the virulent *A. hydrophila* strain LP-2, LP-3, and YT-1, respectively. The overall survival rates of zebrafish immunized with BSA (control), ORF01609, ORF01830, ORF01839, ORF02943, ORF03355, and ORF03641 were 16.7, 68.8, 62.5, 83.3, 77.1, 66,7, and 70.8%, respectively, when challenged with *A. hydrophila* LP-2 (Figure 8A). The overall survival rates of zebrafish immunized with recombinant proteins as previously described order were 16.7, 64.6, 60.4, 75.0, 72.9, 58.3, and 66.7%, respectively, when challenged with *A. hydrophila* LP-3 (Figure 8B). And the overall survival rates of zebrafish were 12.5, 60.4, 58.3, 79.2, 72.9, 70.8, and 64.6%, respectively, when challenged with *A. hydrophila* YT-1 (Figure 8C).The RPS of the fish injected with 3 μg of each recombinant protein (ORF01609, ORF01830, ORF01839, ORF02943, ORF03355, and ORF03641) were 63.0 ± 6.3, 55.6 ± 6.1, 80.2 ± 4.1, 73.0 ± 7.5, 60.6 ± 14.6, and 65.2 ± 5.4% in *A. hydrophila* LP-2 challenging for 14 days, respectively, as compared to the unimmunized control. The RPS of the fish injected with recombinant proteins as previously described order were 57.3 ± 6.0, 52.6 ± 2.2, 70.1 ± 6.4, 67.5 ± 8.2, 50.1 ± 6.3, and 60.0 ± 5.4% in *A. hydrophila* LP-3 challenging for 7 days, respectively. The RPS of the fish injected with recombinant proteins were 53.7 ± 3.6, 51.3 ± 7.4, 74.2 ± 3.9, 69.3 ± 6.7, 65.7 ± 5.1, and 58.4 ± 5.4% in *A. hydrophila* YT-1 challenging for 7 days, respectively. Thus, RPS across all of the groups injected with recombinant proteins was >50%, indicating that these proteins, especially ORF01839 and ORF02943, may be promising candidates for *A. hydrophila* vaccine development.

## Discussion

Secreted proteins play important roles in bacterial virulence during host infection, and have been previously proposed as potential vaccine candidates (30, 31). However, the biological functions and protective efficacies of these proteins remain largely unknown. In addition, iron availability is an important environmental factor for bacterial reproduction, which is always trace available ferrous iron in the natural environment or in the host, may form part of an effective bacteriostatic strategy (32). Thus, iron-regulated proteins may contribute to bacterial virulence, and have been shown to be effective vaccine candidates against pathogenic infection (33). Meanwhile, component vaccines are highly specific and effective in protecting the immune system, but often fail to protect the host well when pathogenic microorganisms mutate (34). Moreover, due to the large number of pathogenic bacteria components, a lot of manpower, and resources are needed to develop new vaccines. Thus, the protein vaccine approach is more prefer for developing and researching new vaccines from a proteomic perspective (35).

To better understand secreted protein behavior in *A. hydrophila* LP-2 in response to iron starvation; to investigate the immune response that these proteins provoked in fish; and to test the efficacy of these proteins as a vaccine, we isolated the extracellular protein fraction in an iron-limited medium (i.e., treated with DIP) and in an untreated LB medium with the trichloroacetic acid (TCA) precipitation method (36–38). The extracellular proteins differentially expressed between groups were compared using iTRAQ label-based quantitative proteomics in combination with high resolution mass spectrometry. The proteomics analysis identified 341 proteins, 9 of which were upregulated in response to iron starvation, and 24 of which were downregulated.

Our proteomics results also indicated that only 10–30% of all differentially expressed proteins were extracellular proteins. However, unavoidable contamination from highly abundant cytoplasmic proteins might have affected the quality of the extracellular fraction. In addition, some proteins are predicted to be in multiple subcellular locations and might be secreted to extracellular space. One such protein was exodeoxyribonuclease III ORF01296, which was predicted to be located in both the extracellular space and in the cytoplasm. Indeed, it is unknown whether cytoplasmic proteins originate from cell lysis or are exported by a so-far unknown mechanism (39). In bacteria, some cytosolic proteins, such as glyceraldehyde-3-phosphate dehydrogenase (GAPDH), ornithine carbamoyltransferase, and α-enolase, have been reported to be secreted proteins, which was consistent with our proteomics results (40–42). Beside these, the secretion of bacterial outer membrane vesicles (OMVs) may also contribute components to the extracellular fraction, which include diverse proteins from different cellular locations (43). Thus, it is complicated to determine the exact components of excreted proteins. We therefore focused on the biological functions and immunoprotective efficacies of the iron-related proteins in the extracellular fraction.

We found that various biological processes, including carbohydrate metabolism, amino sugar metabolism, catabolic metabolism, and chitin metabolic processes, were enriched in the proteins downregulated in response to iron starvation. The downregulation of these proteins might reduce iron consumption, as these proteins use iron as a cofactor (44, 45). We also found that some extracellular proteins associated with chitinase metabolism, including ORF03511, ORF03513, ORF02296, and ORF03889 (homologous to AHA_0979, AHA_0977, AHA_2363, and AHA_0610 (chitin binding protein), respectively, in *A. hydrophila* ATCC 7966) were downregulated in response to iron starvation. Since chitin is a polymer of N-acetylglucosamine and glucosamine residues, and because it forms complexes with transition metal ions including ferric iron, this observed decrease in chitinase metabolism in response to iron starvation is reasonable (46). Proteins related to peptidase activity, the cellular response to stress, and the ABC transport processes were upregulated; these may improve bacterial survival in iron-limited environments by increasing bacterial virulence or adaptability (47).

We validated our proteomics results with Western blotting and qPCR. Six selected genes encoding proteins differentially expressed in response to *A. hydrophila* LP-2 iron starvation (*orf01609, orf01830, orf01839, orf02943, orf03355*, and *orf03641*) were cloned, overexpressed, and purified. Polyclonal antibodies specific to each gene were produced by immunizing mice. Western blots showed that these proteins were upregulated in response to iron starvation, which is consistent with our proteomics results. This indicated that our proteomics results were reliable. Moreover, we analyzed the homologies of these six proteins among twelve *A. hydrophila* strain genomes and found that most of them have considerable high identities (>80%), although the identities of ORF02943 in several strains are slightly low (>30%, Figure S1). We also compared the cross-immunogen reaction of these antigens among *A. hydrophila* LP-2, *A. hydrophila* ATCC 7966, and other popular fish pathogens such as *V. alginolyticus, E. tarda, V. parahaemolyticus*, and *V. fluvialis* by western blotting (Figure S2). Our results showed that many of antibodies against candidate proteins in *A. hydrophila* LP-2 cross-reacted with *A. hydrophila* ATCC 7966 except for ORF02943 and ORF03355, which indicate these specific antibodies plays role in the cross-immune protection among *A. hydrophila* strains. Besides these, some antibodies against antigens such as ORF01609, ORF01830, ORF01839, and ORF03641 have cross-reaction with other fish pathogens. The immunogenicity and immunoprotection properties of these candidate proteins against *A. hydrophila* spp. and other fish pathogens should be further investigated. qPCR analyses also indicated that most of selected genes were upregulated, suggesting a high correlation between mRNA expression and protein expression. These results indicated that the upregulated iron-related proteins might be involved in iron homeostasis or bacterial virulence, and that they had potential immuoprotective properties.

Some of the selected proteins (or their homologs) have been suggested as potential vaccine candidates or virulent effectors. ORF01839 (highly homologous to FecB in *A. hydrophila* ATCC 7966) is a periplasmic iron-binding protein that may affect the iron (III) dicitrate transport system by mediating iron uptake during invasive infections (48). The upregulation of this protein in response to iron starvation, as observed here, might increase the likelihood of bacterial survival by increasing iron uptake. ORF01839 is also homologous to the siderophore-binding protein FhuD in *Staphylococcus aureus*, which belongs to iron regulated lipoproteins (IRLPs) and was reported to involve in the uptake of iron through siderophore (49). IRLPs may be the dominant immunobiologically active compounds in *S.aureus* and induce cytokine by Toll-like receptors (TLRs) (50). Moreover, it was reported that FuhD2 vaccination could evoke protective immunity against clinical *S. aureus* in infection mice by producing functional antibodies to mediate opsonophagocytosis, and has recently been described as a promising vaccine candidate (51, 52). ORF01609 is a serine protease (homologous to Ahe2), and was reported to be a type III secretion system virulence effector in both *A. hydrophila* J-1 and in *A. salmonicida* (53, 54). This protein is a multifunctional enzyme and a key factor of physiological and pathological inflammation which usually involved in the production of pro-inflammatory cytokines and the activation of immune cells such as macrophages (55, 56). Here, the deletion of *orf01609* significantly reduced the virulence of *A. hydrophila* LP-2 in zebrafish, suggesting that this protein is also an important virulence effector in *A. hydrophila*. Additionally, previous researches reported that extracellular serine protease Esp inhibits biofilm formation of *S. aureus* by cleaving autolysin (Atl)-derived murein hydrolases (57). However, the deletion of *orf01609* showed not difference in biofilm formation in this study, which suggest this protein may play more role on the invasion and that would helpful for the bacterial iron uptake from host.

ORF03641 is a hemagglutinin/elastase, which acts as an extracellular zinc-containing metalloendopeptidase. Unlikely extracellular zinc protease in *V. cholerae* participates in biofilm development, this protein may play an important role in bacterial infection by degrading various plasma proteins in the host, including Fe-containing proteins such as immunoglobulin and lactoferrin, thereby increasing the iron resources available to the pathogen (58–60). It was reported that neutrophil elastase can subvert the immune response by cleaving Toll-like receptors and cytokines in pneumococcal pneumonia, which indicate ORF03641 may play similar role on the host immune response (61). Moreover, previous research showed that immunization with elastase peptide reduced experimental lung infections caused by *Pseudomonas aeruginosa* and *Burkholderia cepacia*, indicating that this protein has immunogenic properties (62).

ORF03355 is an outer membrane autotransporter that contains a β-barrel structure according to the humongous analysis. Autotransporters have been shown to have immunoprotective effects against diverse pathogenic bacteria, including *Bordetella pertussis, Haemophilus ducreyi*, and *Edwardsiella tarda* (63–65). Previous research also showed that an outer membrane autotransporter regulates a component of the host inflammatory response, so that *Helicobacter pylori* can fine-tune the host immune response according to the expression of the outer membrane protein (66). That indicates ORF03355 may also play important role on the host immune response. ORF02943 and ORF01830 are hypothetical proteins predicted to be located either on the outer membrane or in the extracellular space. Here, the *orf01830* mutant affected the virulence of *A. hydrophila* LP-2, suggesting that the encoded protein plays a role in bacterial invasion. However, the immuoprotective characteristics of the proteins upregulated in *A. hydrophila* in response to iron starvation remain poorly understood.

To investigate the potential utility of these selected proteins as vaccine candidates, we evaluated the immune response that these proteins provoked in fish as well as their immunoprotective properties. Although immunization with ORF02943 slightly decreased MHCII expression in zebrafish, immunization with the recombinant proteins largely upregulated the six immune-related genes in zebrafish, as compared to the unimmunized control. ORF03641 immunization was particularly effective, increasing the expression of the selected immune-related genes by 16- to 120-fold. In current years, several researches have reported that outer membrane proteins (OMPs) such as OmpA1, OmpC, and Tdr in *A. hydrophila* affect the immune responses and protective efficacies in fish (14, 67, 68). For example, the rOmpF-immunized *Labeo rohita* showed an up-regulated expression of a proinflammatory cytokine IL-1β and phagocytes activation factor INF-α on day 28 challenge (69). rOmpR in *Aeromonas hydrophila*, for another example, was reported to elicit immune response genes such as lysozyme G, T cell response factors (MHC I, MHC II), and IL-1β in *L. rohita* at early time points post-immunization (70). Similar with the immune response of OMPs, immunization with targeted proteins increased the expression levels of several proinflammatory molecules, such as IL-1β, TNF-α, and IL-8 as well as cytokines IL-10 in this study. Likely outer membrane proteins, these extracellular proteins may be recognized by the pattern recognition receptors on the host cells (69), substantially increased the fish immune response, and their immunoprotective characters should be further explored.

Generally, oral, injection and immersion immunization are the three most popular immunization methods. Despite of the disadvantages of labor-intensive and be more likely to cause damage to fish, intraperitoneal injection immunization is more efficient, effective, and stable. It is by far the most effective method and is widely used in injection model for fish challenge (26, 71). Thus, the vaccine efficacy of these recombinant proteins was evaluated by challenging zebrafish with virulent *A. hydrophila*, after intraperitoneal injection immunization in this study. Our results showed that these proteins may be promising vaccine candidates, as RPS ranged from 55 to 80%. ORF02943 and ORF01839 increased RPS substantially, to 73.0 ± 7.5 and 80.2 ± 4.1% in *A. hydrophila* LP-2 challenging, respectively.

We further investigated whether these recombinant proteins eliciting high immune protection against other *A. hydrophila* pathogen infection could protect zebrafish. Therefore, recombinant proteins were used to immunize zebrafish and then challenged with virulent *A. hydrophila* YT-1 and LP-3 for 7 days. Our results showed that all proteins showed significant protection against *A. hydrophila* infection with considerable RPSs in the range from 50.1 ± 6.3 to 74.2 ± 3.9%, especially for ORF01839 and ORF02943 which RPSs were higher 65%. That indicates these proteins have the potential cross-protective abilities in *A. hydrophila* infection. In general, our results compared the differentially expression of extracellular proteins in iron starvation condition using proteomics method and provided an effective strategy for the discovery of recombinant protein vaccine candidates. Additionally, based on the facts that many described proteins of *A. hydrophila*, especially for OMPs and extracellular proteins, have considerable protective effects in fish, which share similar effect with the results in this study, the current candidate proteins can be combined with them as an effective vaccine against the bacterial pathogen *A. hydrophila* in the future.

## Ethics Statement

This study was approved by Fujian Agriculture and Forestry University Animal Care and Use Committee (Certification Number: CNFJAC0027).

## Author Contributions

XW and YW performed most of the experiments. ZL performed data analyses. YF and XY helped for gene clone. WL and XL designed all experiments. XL and FA wrote the manuscript.

### Conflict of Interest Statement

The authors declare that the research was conducted in the absence of any commercial or financial relationships that could be construed as a potential conflict of interest.

## Supplementary Material

Supplementary Figure 1 Homology comparison of target proteins in different *A. hydrophila* stains. The protein homology of different bacteria is expressed as identity from 0 to 100%, and the corresponding color changes from blue to red.

Supplementary Figure 2 Western blot for cross-immunogen reaction among seven species of bacteria with and without DIP treatment. The protein samples were separated with SDS-PAGE and the Western blotting was performed with anti-ORF01609, anti-ORF01830, anti-ORF01839, anti-ORF02943, anti-ORF03355, and ORF03641, respectively.

Supplementary Table 1 Cloning primers sequences in this study.

Supplementary Table 2 qPCR primers sequences in this study.

Supplementary Table 3 Mutant construction primers sequences in this study.

Supplementary Table 4 Identification of extracellular proteins in *A. hydrophila* LP-2 with DIP treatment by iTRAQ labeling analysis.
