# Supplementary material for: Comparative Extracellular Proteomics of Aeromonas hydrophila Reveals Iron-Regulated Secreted Proteins as Potential Vaccine Candidates
Source: Front Immunol. 2019 Feb 18;10:256. doi: 10.3389/fimmu.2019.00256 (PMC6387970; doi:10.3389/fimmu.2019.00256)
Supplement: Supplementary Figure 1 — Homology comparison of target proteins in different A. hydrophila stains. The protein homology of different bacteria is expressed as identity from 0 to 100%, and the corresponding color changes from blue to red. [file Data_Sheet_1.doc]

**Supplementary Figure S1. Homology comparison of target proteins in different *A. hydrophila* stains.** The protein homology of different bacteria is expressed as identity from 0% to 100%, and the corresponding color changes from blue to red.

**Supplementary Figure S2. Western blot for cross-immunogen reaction among seven species of bacteria** **with and without DIP treatment.** The protein samples were separated with SDS-PAGE and the Western blotting was performed with anti-ORF01609, anti-ORF01830, anti-ORF01839, anti-ORF02943, anti-ORF03355 and ORF03641, respectively.

**Supplementary Figure S1**

**
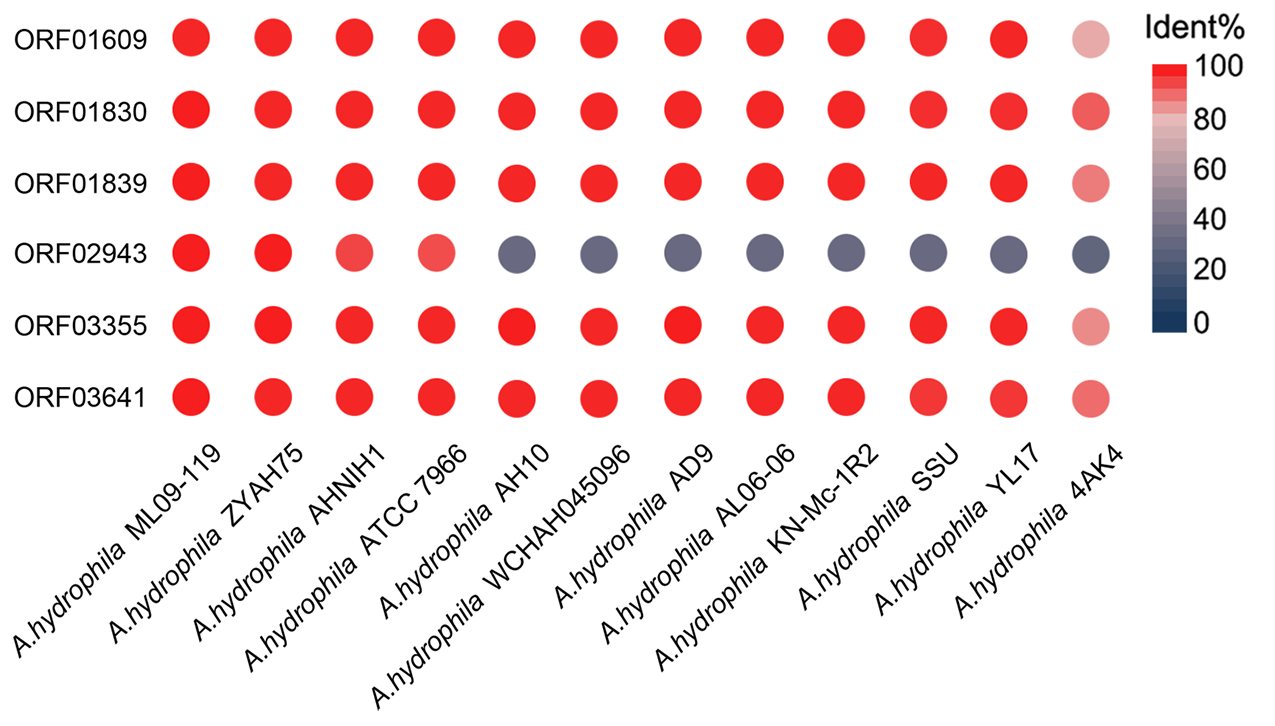
**

**Supplementary Figure S2**

**
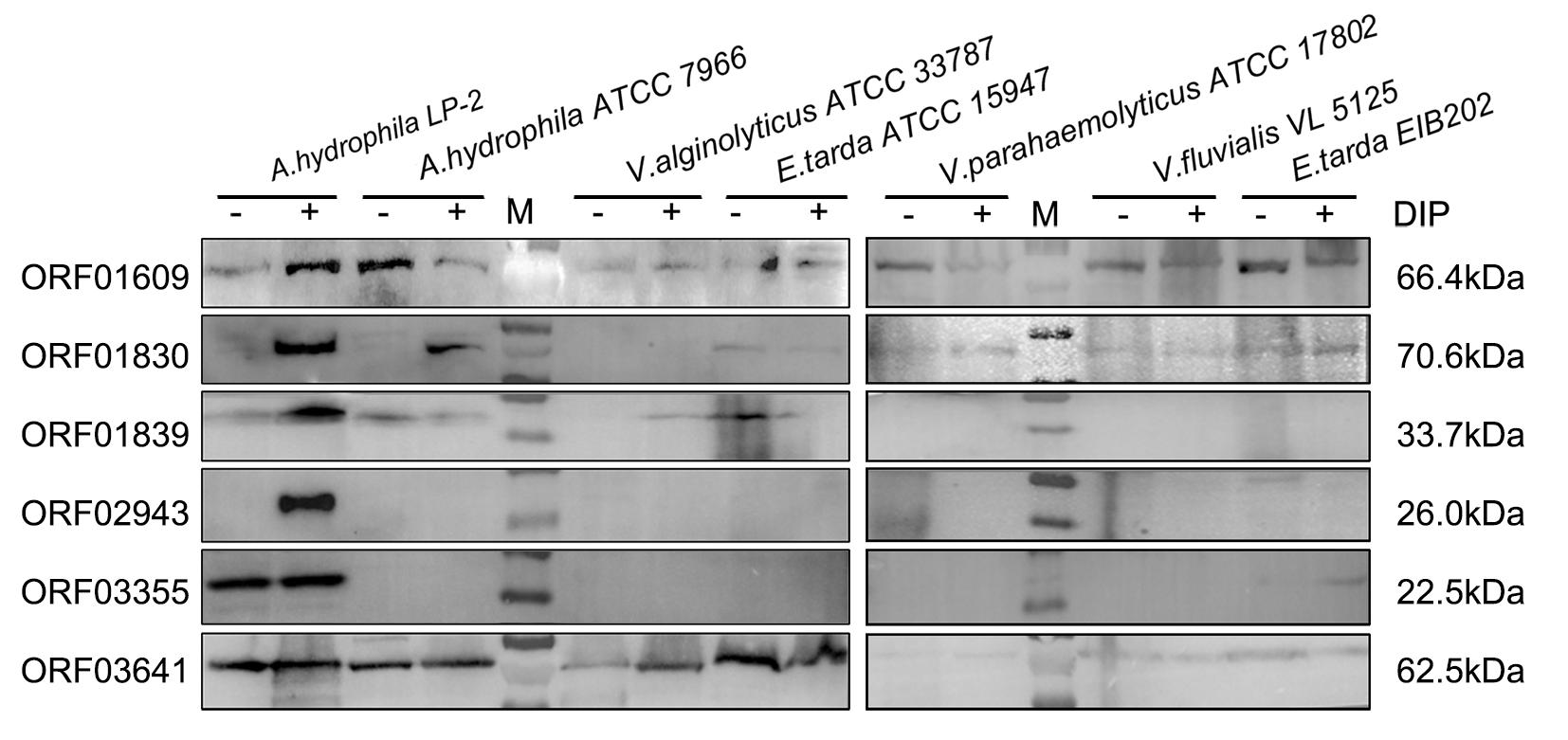
**
