# Supplementary material for: Comparative Extracellular Proteomics of Aeromonas hydrophila Reveals Iron-Regulated Secreted Proteins as Potential Vaccine Candidates
Source: Front Immunol. 2019 Feb 18;10:256. doi: 10.3389/fimmu.2019.00256 (PMC6387970; doi:10.3389/fimmu.2019.00256)
Supplement: Supplementary Table 1 — Cloning primers sequences in this study. [file Table_1.DOCX]

**Supplementary Table S1. Cloning primers sequences in this study**

| **Primer name** | **Primer sequences**（5’→3’） | | **Res.site** |
| --- | --- | --- | --- |
| ORF01609 | Sense | CGGAATTCATGAGAAAAGCATCGTTAGCGTTG | *EcoRⅠ* |
|  | Antisense | CCAAGCTTTGAGCGGGCGGCATCGTG | *HindⅢ* |
| ORF01830 | Sense | GGGAATTCATGAGAAAAACCGTACTGG | *EcoRⅠ* |
|  | Antisense | CCCAAGCTTTTATTTTTCGGTGATCAGC | *HindⅢ* |
| ORF01839 | Sense | CGGAATTCATGAAGATCTCCTCTCCCCTGGC | *EcoRⅠ* |
|  | Antisense | CTAAGCTTTTATTGGGTGATGGGGGCCG | *HindⅢ* |
| ORF02943 | Sense | CGGGATCCCAGATGCACCGCTATTTCC | *BamHⅠ* |
|  | Antisense | CCCAAGCTTCTCCTCAATGGTTGGGATGGG | *HindⅢ* |
| ORF03355 | Sense | CGGAATTCATGAATAAGGCTGCACTTTCAGTTC | *EcoRⅠ* |
|  | Antisense | CCCAAGCTTGAAGCTGTACTTCACACCCAGG | *HindⅢ* |
| ORF03641 | Sense | CGGAATTCCGGATGAACAAAGTCTATT | *EcoRⅠ* |
|  | Antisense | CCAAGCTTTCAGTAGCTGGCCTTGAGGGTC | *HindⅢ* |

Note: All genes were cloned into pET32a vector.
