# Supplementary material for: Comparative Extracellular Proteomics of Aeromonas hydrophila Reveals Iron-Regulated Secreted Proteins as Potential Vaccine Candidates
Source: Front Immunol. 2019 Feb 18;10:256. doi: 10.3389/fimmu.2019.00256 (PMC6387970; doi:10.3389/fimmu.2019.00256)
Supplement: Supplementary Table 2 — qPCR primers sequences in this study. [file Table_2.DOC]

**Supplementary Table S2. qPCR primers sequences in this study**

| **Gene** | **Primer sequences**（5’→3’） |
| --- | --- |
| β–actin | For zebrafish studies in gene expression levels  F:ATGGATGAGGAAATCGCTGCC R:CTCCCTGATGTCTGGGTCGTC |
| Lyz | F:GATTTGAGGGATTCTCCATTGG R:CCGTAGTCCTTCCCCGTATCA |
| MHC I | F:GGAGTTCACCTTGCTTATGC R:CCCTCTGACCCATTCTTGT |
| MHC II | F:TGACTCAACTGTCCGTGATA R:CCATTAGCCATCTCCATAGTG |
| IL-1β | F:TGGACTTCGCAGCACAAAATG R:GTTCACTTCACGCTCTTGGATG |
| IL-8 | F:GTCGCTGCATTGAAACAGAA R:CTTAACCCATGGAGCAGAGG |
| IL-10 | F:TCACGTCATGAACGAGATCC R:CCTCTTGCATTTCACCATATCC |
| IL-15  TNF-α | F:ACAGAGGAAGAAGCCTACAG R:GCGATGAAGACGAGAAAGAG  F:ATAAGACCCAGGGCAATCAAC R:CAGAGTTGTATCCACCTGTTAAATG |
| *16SrRNA* | For *A. hydrophila* LP-2 studies in gene expression levels  F:GGAGCAAACAGGATTAGATACC R:GGAAGCCACGTCTCAAGGA |
| *orf00614* | F:AAGAACCTCACCGGCACCAT R:GCAGCACCGCCAGATAGTAGAT |
| *orf01609* | F:AGCTGAAGGCATTCTGGC R:TTAGCGTTGGCAATCTCG |
| *orf01830* | F:CGGTCTGGATCTGGATGTGC R:CGCCTCTTCGGAGTATTCGT |
| *orf01839* | F:GGTGGTGGTACTGAGCGAAAT R:CTTCTCCAGGTTAGGGTTGTCC |
| *orf02793* | F:CCGCTGTTATCCGTTGTTATCG R:GGTGAACTCCTTGCCGATGC |
| *orf02943* | F:GACATCCCTCCAATCACAGA R:CGGTTACCTCATCCCACTT |
| *orf03355* | F:TCGGCGGCAAGGATAGCAAC R:CGTAGAAGTTGGAACCGAAGC |
| *orf03641* | F:TCTATTTGGCCGTGGTATTGG R:CCCGCACCTGACGAAACT |
| *orf04443* | F:TGTCCGGGCGCTATCAG R:TGGCATCCAGGGTGTCG |
| *orf04406* | F:CCAAGCCTGAGCACTACCC R:CAGCCGAATCCAGCACC |
| *orf03984* | F:CAATGACAGTGAGGCTTACC R:CACTCGTCGGAGTCCTTGTT |
| *orf03513* | F:CCACATCCTCTACGGTTTCA R:TCAGGTTGCCGAAGTTGC |
