# Supplementary material for: Comparative Extracellular Proteomics of Aeromonas hydrophila Reveals Iron-Regulated Secreted Proteins as Potential Vaccine Candidates
Source: Front Immunol. 2019 Feb 18;10:256. doi: 10.3389/fimmu.2019.00256 (PMC6387970; doi:10.3389/fimmu.2019.00256)
Supplement: Supplementary Table 3 — Mutant construction primers sequences in this study. [file Table_3.DOC]

**Supplementary Table S3. Mutant construction primers sequences in this study**

| **Primer name** | | **Primer sequences**（5’→3’） |
| --- | --- | --- |
| *orf01609*-1F | Sense | CATGAATTCCCGGGAGAGCTCCTGGCGTTGATCAAATAGCTCTC |
| *orf01609*-2R | Antisense | GACTCCTTGTAATAAACTCCATCTTATATCGCACCG |
| *orf01609*-3F | Sense | GGAGTTTATTACAAGGAGTCACAAGATGAAACACAG |
| *orf01609*-4R | Antisense | CGATCCCAAGCTTCTTCTAGAGATGGTCAATGAGGATGGCTG |
| *orf01609*-P7 | Sense | TGGAGATGTGCCTCTACGA |
| *orf01609*-P8 | Antisense | CGACAGGGAAGGTGATTTG |
| *orf01830*-1F | Sense | CATGAATTCCCGGGAGAGCTCAGATGCAAAGCCGCATCG |
| *orf01830*-2R | Antisense | GAGTGGCTGATTCTCACCATATGTTTGATTTTATTATGA |
| *orf01830*-3F | Sense | ATGGTGAGAATCAGCCACTCGCTGAATAGAAGA |
| *orf01830*-4R | Antisense | CGATCCCAAGCTTCTTCTAGAAACATGGATCTCGCCTCCG |
| *orf01830*-P7 | Sense | GCCGCCTGTTCACTCACCCA |
| *orf01830*-P8 | Antisense | CGTTTGCCCACCCGTTCA |
| *orf03641*-1F | Sense | CATGAATTCCCGGGAGAGCTCGCAGCCACTGGGTCAGGTAG |
| *orf03641*-2R | Antisense | CAGTCCTTAAGTCGATGAAGATTACACGTC |
| *orf03641*-3F | Sense | CTTCATCGACTTAAGGACTGTAAATAGAGAGGGAGCGCC |
| *orf03641*-4R | Antisense | CGATCCCAAGCTTCTTCTAGAATCCAGCTCGAGCTGCTCTATC |
| *orf03641*-P7 | Sense | TGGTTGTAACCGATGACGC |
| *orf03641*-P8 | Antisense | GCTGCTCTATCGCTCACC |
